# Fabrication and characterization of shape memory polyurethane/GNP/MWCNTs nanocomposite thin-films with enhanced UV resistance

**DOI:** 10.1038/s41598-026-44601-6

**Published:** 2026-03-24

**Authors:** Sivanagaraju Namathoti, Abdullah A. Elfar, Venkata Dinesh Avvari, Santosh Kumar Sahu, Borhen Louhichi, It Ee Lee, Qamar Wali, P. S. Rama Sreekanth

**Affiliations:** 1 School of Mechanical Engineering, VIT-AP University, Inavolu, Amaravati, 522337 AP India; 2 Department of Mechanical Engineering, College of Engineering, Imam Mohammad Ibn Saud Islamic University (IMSIU), Riyadh, 11432 Saudi Arabia; 3 Engineering Sciences Research Center (ESRC), Deanship of Scientific Research, Imam Mohammad Ibn Saud Islamic University (IMSIU), Riyadh, 11432 Saudi Arabia; 4 Faculty of Artificial Intelligence and Engineering, Multimedia University, Cyberjaya, 63100 Malaysia; 5 Centre for Smart Systems and Automation, COE for Robotics and Sensing Technologies, Multimedia University, Cyberjaya, 63100 Malaysia

**Keywords:** SMPU thin-films, GNPs, MWCNTs, UV irradiation, Mechanical, Thermal, Shape recovery performance, Engineering, Materials science, Nanoscience and technology

## Abstract

Polymer films provide multifunctional platforms for diverse technological applications such as protective coatings, miniaturized sensors, biomedical devices, and energy storage systems. Shape memory polyurethane (SMPU) is a smart polymer capable of responding to external stimuli; however, its moderate mechanical strength and limited shape recovery efficiency restrict broader utilization. In this study, SMPU thin-films were independently reinforced with 1 wt% graphene nanoplatelets (GNPs) and multi-walled carbon nanotubes (MWCNTs) using the solvent-casting method. The resulting nanocomposite thin-films were comprehensively characterized to evaluate their thermal stability, mechanical performance, structural features, and shape recovery behavior, as well as the influence of nanofillers on their UV resistance under accelerated irradiation. The incorporation of GNPs and MWCNTs markedly enhanced the mechanical, thermal, shape recovery, and UV-resistant properties of SMPU. After 24 h of UV irradiation, tensile strength increased by 69% in GNP-reinforced and 72% in MWCNT reinforced thin-films. Notably, the shape recovery rate accelerated by 10–20%. Moreover, owing to the superior UV absorption and radical-scavenging abilities of both nanofillers, the nanocomposite thin-films exhibited lower photooxidative index values compared to pure SMPU. Hence, the findings of this study contribute to the development of high-performance SMPU-based nanocomposites for various potential applications.

## Introduction

 Shape memory polymers (SMPs) are smart materials that respond to external stimuli like heat, pH, light, electricity, or water. Of these, shape memory polyurethane (SMPU) has been investigated extensively for thermally induced shape memory. SMPU is composed of soft and hard segments: the hard segments serve as physical net points to pin the original shape, whereas the soft segments store and release stress on deformation and recovery^1^. Although SMPU offers advantages such as high elasticity and low density, its practical use is limited by low mechanical strength and inadequate shape recovery performance. To counter these limitations, nanofillers like graphene (GNP) and multiwalled carbon nanotubes (MWCNTs) are added to enhance energy storage and mechanical properties^2,3^. Such reinforcements adequately strengthen SMPU, and its applications are extended to other areas. Moreover, in films, moisture permeability is seen to exhibit a striking contrast above and below the glass transition temperature (T_g_), a feature of interest in clothing applications.

Polymer films have gained significant attention due to their lightweight nature, flexibility, and ability to impart functional properties without altering the bulk characteristics of the material. They are essential in applications requiring surface modifications, barrier protective coatings^4,5^, miniaturization, and cost effectiveness, and render them extremely desirable sensors^6,7^, biomedical devices^8,9^, energy storage and conversion^10–12^. Among these, SMPU films have gained prominence as they not only inherit the inherent virtues of polyurethanes, such as high elongation, biocompatibility, and low density, but also acquire shape memory function that enables these materials to revert to their original shape upon actuation of external stimulus. Such smart properties and controllable mechanical, thermal, and interfacial attributes render the SMPU films of particular importance for potential use in actuators, flexible electronics, protective coatings, and biomedical applications, including drug delivery devices, wound dressings, and tissue engineering scaffolds. Moreover, when reinforced with nanofillers such as GNPs and MWCNTs, SMPU films exhibit remarkable enhancements in their functional performance.

Considering the past research on graphene-filled polyurethane (PU), Zarghami et al.^13^ fabricated PU nanocomposite films containing varying concentrations of GNPs using the solvent casting method. They reported improvements in the mechanical, thermal, and shape recovery properties of the films. Similarly, Sofla et al.^14^ investigated the effects of reduced graphene oxide (RGO) and RGO grafted on polycaprolactone (PCL) on polyurethane and reported enhanced shape recovery ratios owing to improved thermal conductivity at a temperature of 60 °C. Additionally, Babaei et al.^15^ fabricated polyurethanes based on PCL with varying molecular weights of PCL and reported that the incorporation of graphene, along with a higher molecular weight of PCL, enhanced shape fixity and recovery by inhibiting the mobility of PCL chains and transmission of stress in rigid domains.

Considering the previous research on MWCNTs as a filler in PU, Russo et al.^16^ synthesized TPU nanocomposite films containing MWCNTs and noted an enhancement in tensile properties. Chilito et al.^17^ explored the impact of MWCNTs on thermal, viscoelastic, and electrical properties of thermoplastic polyurethane (TPU). According to their results, the T_g_ was increased while the melting temperature was reduced with increases in conductivity and storage modulus. Wang et al.^18^ produced MWCNT/TPU nanofiber films by electrospinning, and the greater the content of MWCNT, the higher the conductivity and sensing ability. Additionally, Namathoti and Wang et al.^19^ developed SMP/MWCNT nanocomposites and reported significant improvements in the mechanical, thermal, and shape-recovery properties of the SMP.

Moreover, in view of the previous work of UV on polyurethane, Rosu et al.^20^ reported the infrared spectral changes and yellowing of polyurethane caused by UV radiation. Goodwin et al.^21^ examined the UV-induced degradation of reduced graphene/TPU nanocomposites, which assessed the impact of few-layer graphene on polymer degradation and graphene release from the composite surface. Also, Bhargava et al.^22^ investigated the progress of a waterborne aliphatic PU-based coating subjected to accelerated UV, water (WT), and thermal (TH) aging for 1000 h, with periodic assessments of coating durability every 200 h. Similarly, Namathoti et al.^23^ prepared 4D printed SMPU/MWCNT nanocomposites with 0.25-1.0 wt% MWCNTs and discussed the response to UV-B exposure for up to 250–1000 h, and the enhanced mechanical, thermal, and shape recovery characteristics. Furthermore, Adak et al.^24^ studied the enhancement of the weather resistance and helium gas barrier property of TPU films using graphene and described that there is a remarkable improvement in tensile strength and stiffness of nanocomposite films with an increase in graphene content.

Although graphene and MWCNT-reinforced polyurethanes have been widely studied for mechanical and thermal enhancement, limited research has focused on solvent cast SMPU thin-films, particularly under UV exposure conditions. Furthermore, while the UV-shielding behavior of carbon nanofillers is well documented in general polymers, their influence on the retention of shape memory functionality, including recovery ratio, and structural stability under irradiation, remains insufficiently explored.

In this context, the present study aims to (i) fabricate SMPU thin-film nanocomposites reinforced with GNPs and MWCNTs via solvent casting, (ii) systematically evaluate their thermal, mechanical, and structural properties, and (iii) quantitatively investigate the effect of UV exposure on both material degradation and shape memory performance. By correlating photooxidative index, crystallographic ordering, and glass transition behavior with functional recovery characteristics, this work provides a comprehensive understanding of durability function relationships in UV-exposed SMPU thin-films for a variety of potential applications.

## Materials and methods

### Materials

SMPU granules from a semi-crystalline material sourced from SMP Technologies, Japan (pellet type: MM-6520), as the matrix. Dimethyl Formamide (DMF) was purchased from National Scientific & Products India. Amine-functionalized MWCNTs with purity ~ 99%, length > 10 μm, inner dia. 5–10 nm, outer dia.10–20 nm, and NH_2_ ratio 2–5% from Shilpa Enterprises. GNPs with purity ~ 99%, number of layers 5–6, and bulk density 2.2 g/cm^3^ from Shilpa Enterprises. The solutions were prepared using a solution casting method with DMF as the solvent.

### Solution casting methods

**Fig. 1:**
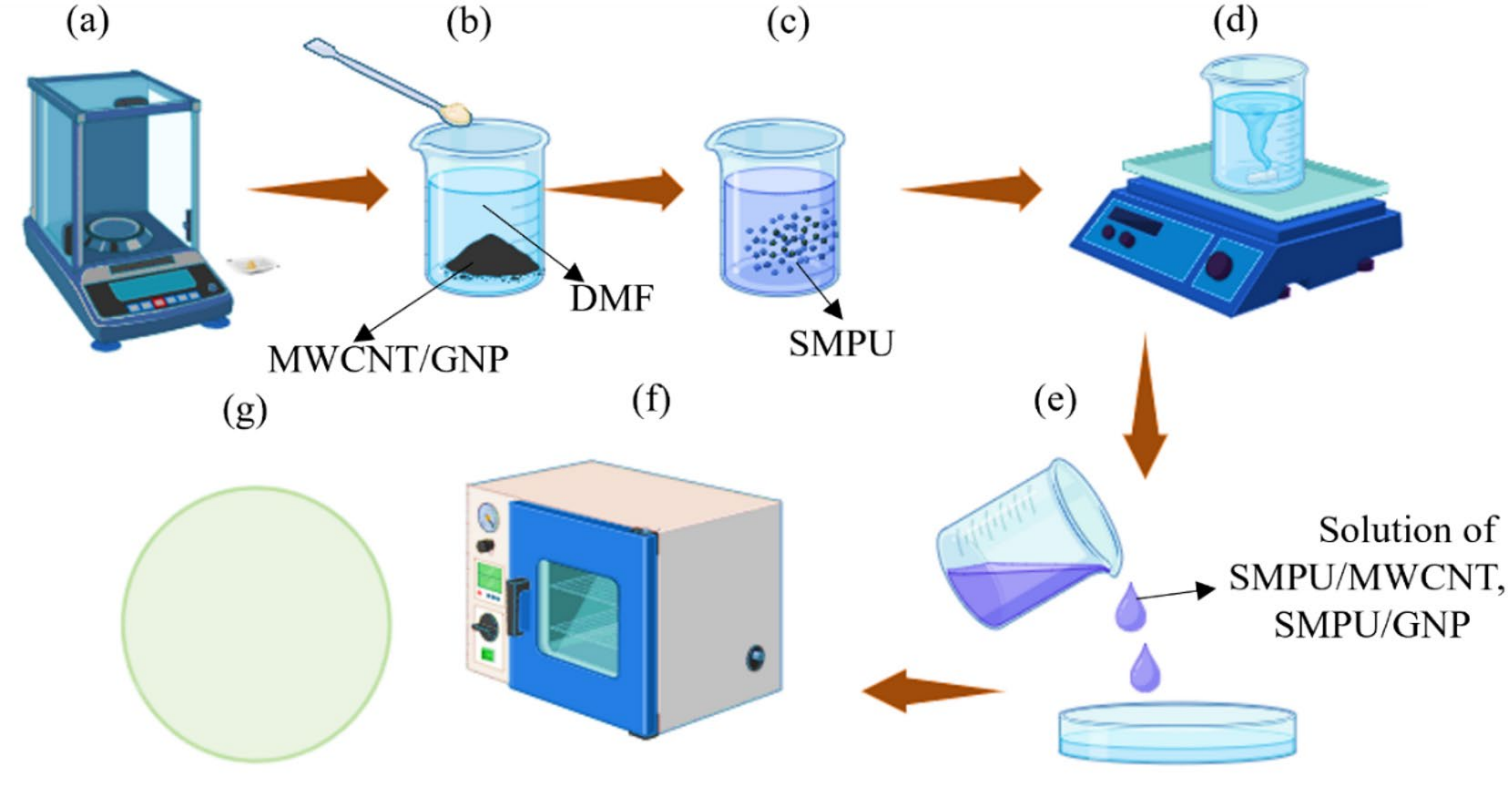
Solution casting process for making SMPU reinforced thin-films.

Table 1 summarizes the solvents and processing parameters employed in the fabrication of films via the solvent casting method, as schematically illustrated in Fig. 1.

**Table 1:** Preparation parameters of the films.

Material/filler	Solvent	Wt.% of filler	Temperature (°C)	Curing time (hrs)	Film thickness (µm)
SMPU	DMF	0	50–60	4	40 ± 5
SMPU/GNP	DMF	1	60–80	6	55 ± 5
SMPU/MWCNT	DMF	1	60–80	6	50 ± 5

### Preparation of SMPU and its nanocomposite thin-films

Fabrication of thin-films with SMPU and its nanocomposites (SMPU/GNP and SMPU/MWCNT) entails a carefully coordinated solvent casting approach. In the beginning, the base SMPU pellets were dissolved in DMF at an 18/82 wt%/vol. ratio under continuous stirring using a magnetic stirrer for 2 h at 50–60 °C to obtain a homogeneous polymer solution. For the nanocomposite films, either the MWCNTs or the GNPs are first further dispersed into DMF by sonication for 60 min before being added to the SMPU solution. The temperature is then raised to 60 °C, and the mixture is stirred for another 1 h to provide good dispersion of nanofillers in the polymer matrix.

The prepared solutions are used in solution casting to form the films, where they are poured onto clean glass or PET substrates. The solutions are then spread uniformly to obtain films of 40–60 μm thickness. The cast films finally go to a vacuum oven for solvent evaporation and curing. Curing of pure SMPU films takes place at 50 °C for 4 h, with a resulting film thickness of about 40 ± 5 μm. Similarly, the procedure was performed for nanocomposite films. The thickness of the final films were SMPU/GNP is 50 ± 5 μm and SMPU/MWCNT is 50 ± 5 μm.

**Fig. 2:**
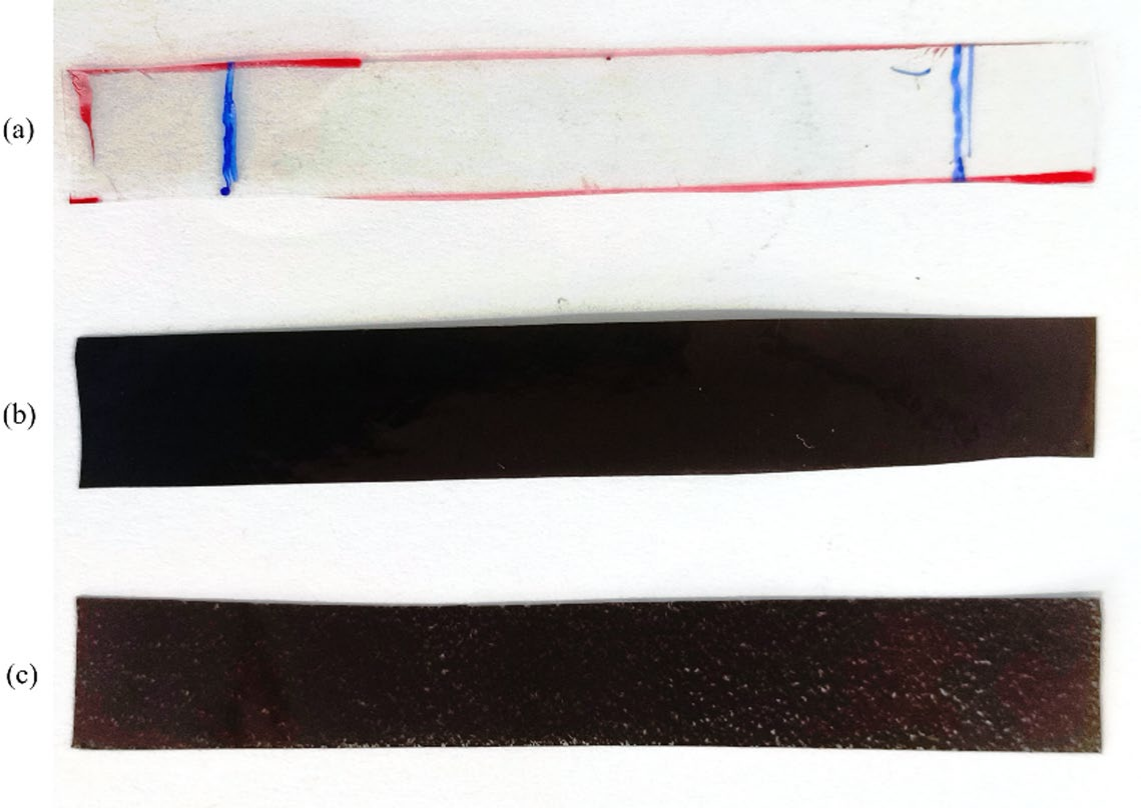
SMPU and its nanocomposite films: (**a**) Pure, (**b**) 1 wt% GNP, and (**c**) 1 wt% MWCNT.

The labels in the Fig. 1a–d-likely refer to the various steps in the pure SMPU film formation process, whereas Fig. 1e–g refers to the resultant nanocomposite films. Hence, thin-films of excellent quality having uniform thickness and properties are produced upon solution casting and controlled curing conditions, making them suitable for a host of applications that demand shape memory or enhanced mechanical. Figure 2 shows the produced thin-films after the process.

### UV irradiation

**Fig. 3:**
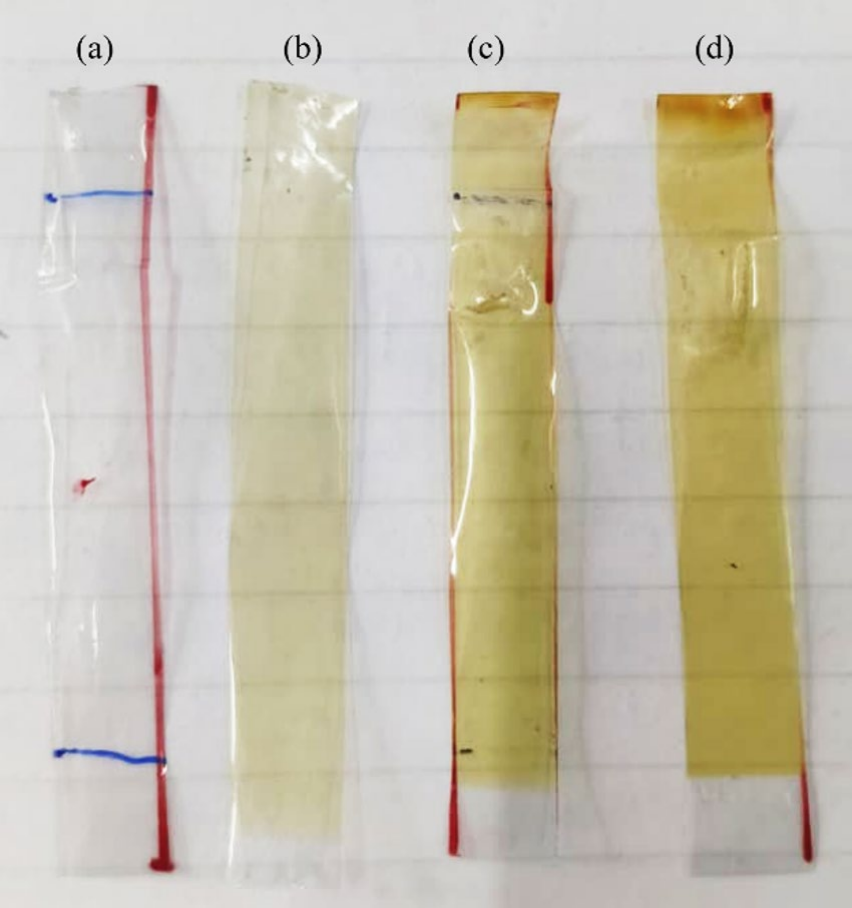
Pure SMPU films at various durations of UV exposure: (**a**) 0, (**b**) 24, (**c**) 48, and (**d**) 72 h.

A set of pure SMPU films and their nanocomposite variants were exposed to UV irradiation using an Accelerated Weathering Tester (AWT), tabletop model, from APPLE Electronics. The UV exposure conditions were maintained at an irradiance of 1.20 W/m² and black-panel temperature 45 ± 5 °C at 340 nm, with exposure times of 24, 48, and 72 h (h) under air atmosphere. Upon irradiation, the films exhibited noticeable discoloration, gradually turning yellow as illustrated in Fig. 3, which is attributed to the photodegradation mechanism^20^.

#### UV dose calculations

The UV irradiance used in this study was 1.20 W/m² at a wavelength of 340 nm. The cumulative UV dose (energy per unit area) was calculated as (1):

1 $${\text{Dose }}({\mathrm{J}}/{\mathrm{m}})^2 = {\text{Irradiance }}({\mathrm{W}}/{\mathrm{m}}^2) \times {\text{Time }}\left( {\mathrm{s}} \right)$$

**For 24 h of exposure**: 1.20 × (24 × 3600) = 103,680 J/m².

Similarly, the cumulative UV doses for different exposure durations are summarized in Table 2.

**Table 2:** UV dosage per unit area.

Time (h)	UV dose (J/m²)
24	1.036 × 10⁵
48	2.073 × 10⁵
72	3.110 × 10⁵

The cumulative UV dose ranged from 1.036 × 10⁵ to 3.110 × 10⁵ J/m², ensuring controlled accelerated aging conditions.

### Experimental characterisation

All material characterizations were performed on both pure SMPU and its reinforced thin-films. Differential Scanning Calorimetry (DSC) analysis was conducted using a Perkin Elmer STA 8000 system. Specimens were scanned from 25 °C to 250 °C at a heating rate of 20 °C/min in the presence of nitrogen gas. Tensile tests were conducted on a Tinius Olsen H10KL Universal Testing Machine according to ASTM D882 at a strain rate of 10 mm/min. FTIR spectroscopy was conducted in attenuated total reflection (ATR) mode on an Agilent Technologies Cary 630 spectrometer. Spectra were obtained in the range of 400–4000 cm⁻¹ for 64 scans per film.

XRD was performed on a Rigaku Benchtop MiniFlex diffractometer with Cu-Kα radiation. Data were collected between 2θ values of 10° and 90° to assess phase composition. The surface morphology of fractured SMPU and its nanocomposite films were investigated using the Carl ZEISS EVO10 (Zeiss Microscopy, LLC, NY, USA). Contact angles were measured on HOLMARC Opto-Mechatronics Pvt. Ltd. equipment by the sessile drop method. Sessile-drop goniometry was employed because it is a convenient and reproducible method of surface-wetting behavior characterization. All the tests were performed at room temperature, and results reported are an average of five specimens per condition.

### Shape memory characterisation

**Fig. 4:**
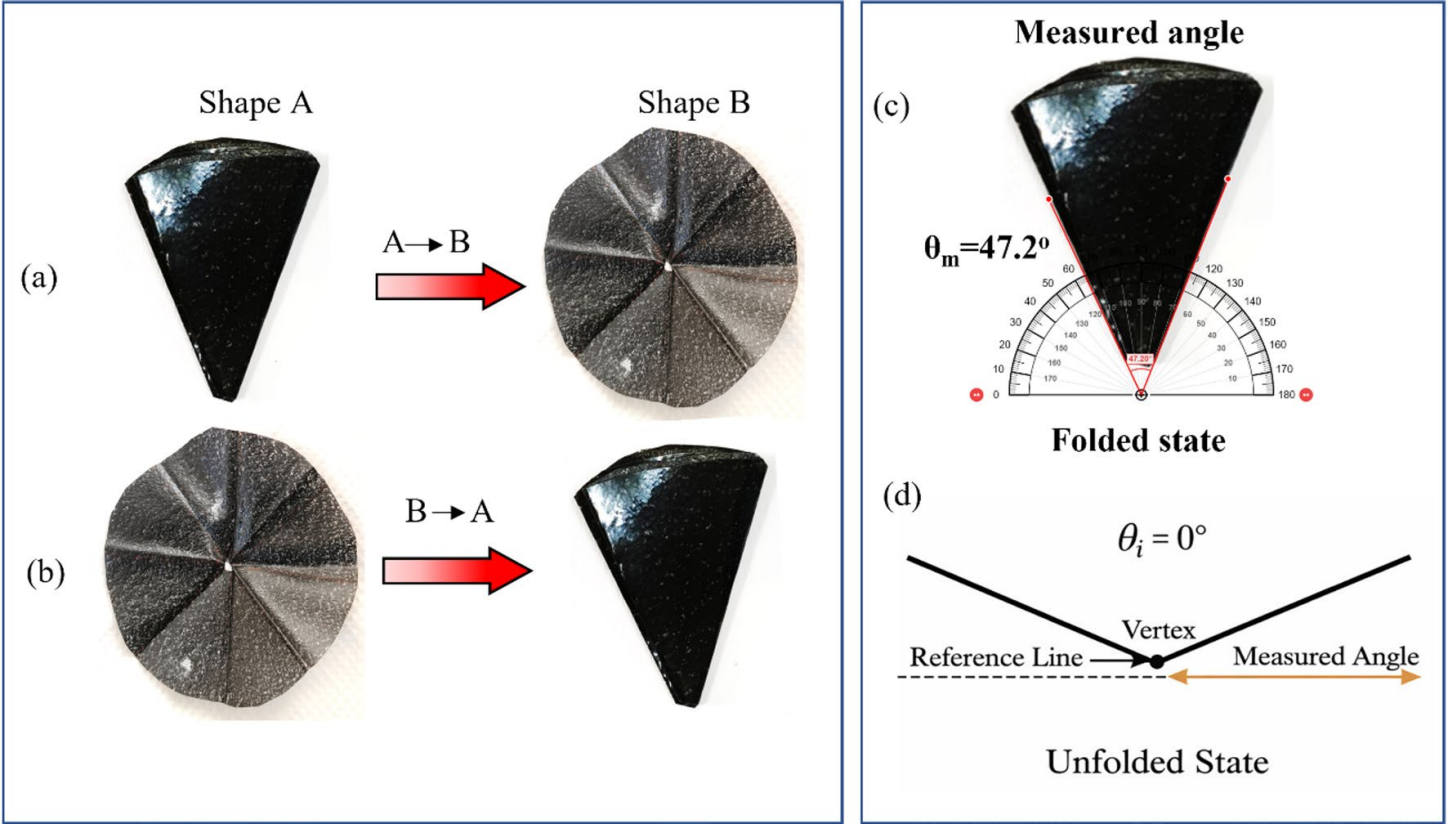
SMPU/MWCNT film during Shape recovery from (**a**) shape A→B, and (**b**) shape B→A transitions, (**c**) approx. angle of folded shape, and (**d**) angle of unfolded shape.

The shape recovery behavior of both the SMPU and their nanocomposite films were assessed under a controlled hot water bath test setup at 75 °C. Two distinct shape transformation sequences were conducted to characterize the recovery deformation behavior of the materials. In the first mode, the films were programmed in shape-A as a folded conical origami shape, and subsequently exposed to the thermal stimulus to recover to the original unfolded conical origami shape, which is stated as shape-B as shown in Fig. 4. In the second mode, the inverse transformation was examined, where the films were initially deformed into shape-B (unfolded conical shape) using the shape training method with hot press, then returned to the folded conical shape (shape-A), following thermal activation.

The recovery process was recorded using a digital camera during thermal activation, and angular measurements were extracted from calibrated images using ImageJ software. Each test was repeated five times, and the reported values represent the average to ensure reproducibility. All measurements were obtained digitally to enhance precision and minimize human error. For shape A→B, the original folded configuration was defined as θ_m_ = 47.2°, and the programmed unfolded shape as θ_i_ = 0°, as shown in the Fig. 4c & d. A recovery ratio of 100% was defined as the condition where the recovered angle returned to approximately θ_i_, with full recovery considered when the recovered angle was within ± 3° of the original geometry.

The variation in shape fixity ratio (R_f_) among pure SMPU, SMPU/GNP, and SMPU/MWCNT films was minimal (within ± 3%), indicating that nanofiller incorporation did not significantly affect the soft-segment freezing mechanism responsible for temporary shape fixation.

## Results and discussion

### Thermal and mechanical properties of GNP and MWCNT-filled SMPU films

**Fig. 5:**
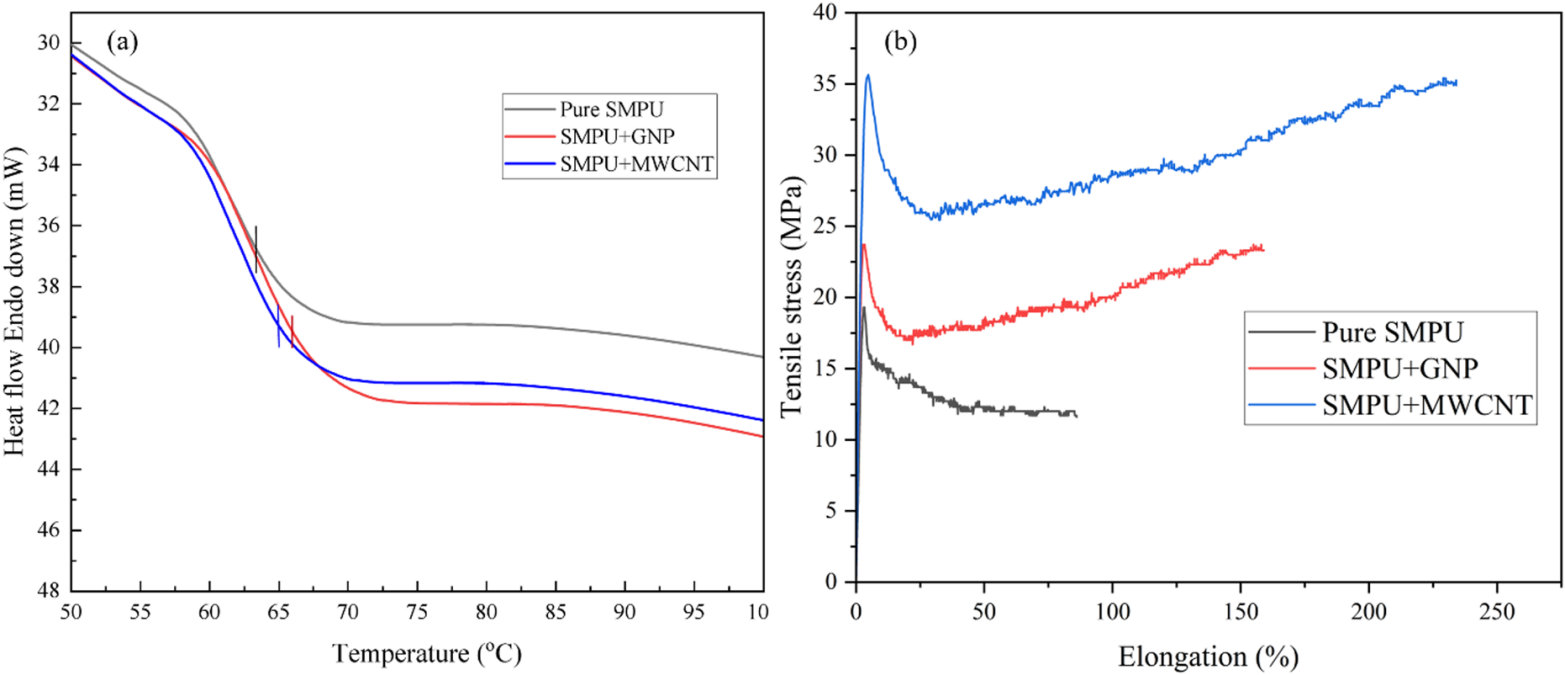
Thermal and mechanical properties of SMPU and its nanocomposite: (**a**) DSC thermograms, (**b**) Tensile test.

The thermal and mechanical behavior of SMPU nanocomposite films was significantly influenced by the addition of GNPs and MWCNTs. The DSC curve, shown in Fig. 5a, reveals that the glass transition temperature (T_g_) of the pure SMPU was 63.5 °C. The addition of 1 wt% GNPs increased the T_g_ to 66.4 °C, while the addition of MWCNT resulted in a T_g_ of 65.6 °C. The T_g_ increases can be due to the strong interfacial interaction between the nanofillers and SMPU chains that restricts the mobility of the soft segments. Furthermore, the increase in T_g_ is attributed not merely to restricted chain mobility, but more specifically to distinct interfacial interactions in each nanocomposite system. In the case of GNP reinforcement, π–π stacking interactions occur between the graphitic basal planes and the aromatic hard segments of SMPU, leading to constrained segmental motion. For the amine-functionalized MWCNT system, the T_g_ enhancement is primarily associated with interfacial hydrogen bonding between the -NH₂ groups on the nanotube surface and the urethane carbonyl (C = O) and N-H groups of the polymer matrix. These interactions are supported by the FTIR results (Fig. 7), which show peak reductions in N-H and C = O band intensities, indicating strong filler–matrix interfacial bonding^13,16,25^.

This restriction on chain mobility also influences the mechanical properties of the nanocomposites, as shown in Fig. 5b. The tensile stress-strain curves indicate that the incorporation of GNPs and MWCNTs significantly enhances both the toughness and strength of the SMPU matrix. The SMPU/MWCNT film showed the highest tensile strength 36 MPa, followed by SMPU/GNP 25 MPa, whereas pure SMPU showed the lowest 17 MPa. The enhanced tensile strength is directly attributed to the enhanced interfacial bonding and load transfer efficiency between the nanofillers and the polymer matrix^13,15^. These high-strength interactions, which were already established by the high T_g_, require more stress to be overcome during deformation.

Apart from strength, the elongation behavior also improved. As observed in Fig. 5b, SMPU had a very low ductility of 90%, while nanocomposites with GNPs and MWCNTs had significantly higher elongation values, 150 and 240%, respectively. However, the simultaneous enhancement in tensile strength and elongation at break is attributed not only to improved interfacial load transfer but also to the ability of well-dispersed nanofillers to act as microcrack deflectors and craze terminators. The high-aspect-ratio nanofillers bridge micro voids and prevent early catastrophic failure, allowing for greater plastic deformation of the matrix. This crack-arresting mechanism, combined with the formation of a mechanically active interphase, results in a strength-ductility synergy at low filler loading^26–28^. This supports the dual function of the nanofillers in limiting the thermal chain mobility while at the same time facilitating the mechanical dissipation of energy on tensile loading, resulting in increased T_g_ and mechanical performance.

**Fig. 6:**
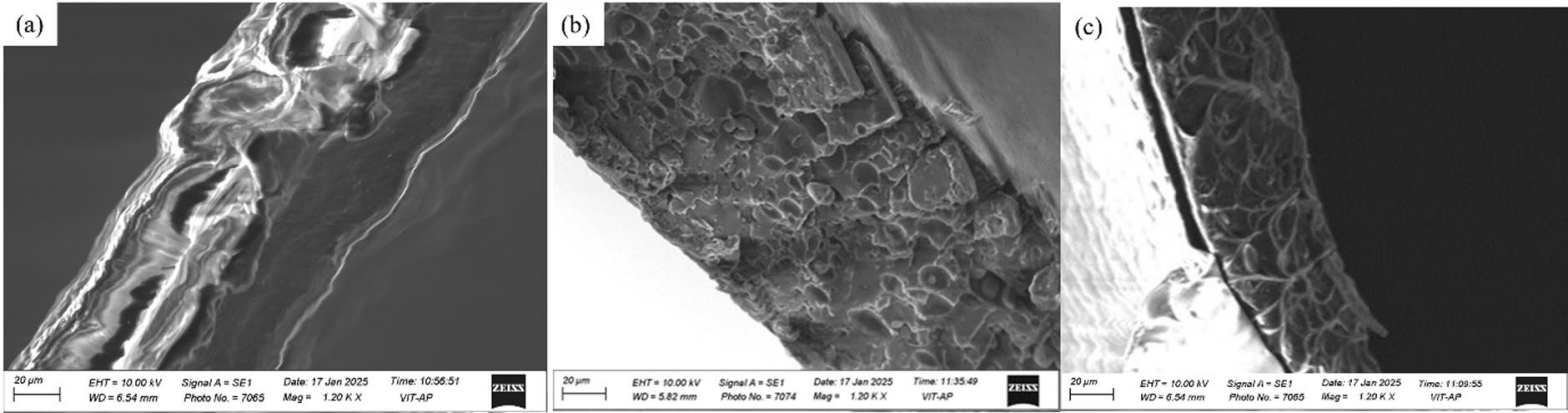
SEM micrographs of SMPU and its nanocomposite thin-films (**a**) Pure SMPU, (**b**) SMPU/GNP, and (**c**) SMPU/MWCNT.

In order to understand the effect of GNPs and MWCNTs on the mechanical strength of the nanocomposite, fractography analysis was performed. Figure 6 illustrates the SEM micrographs of pure SMPU and its nanocomposite films broken in liquid nitrogen. In the case of pure SMPU as shown in Fig. 6a, the surface appears relatively smooth and layered, indicating a ductile fracture with plastic deformation features, which results in lower tensile strength. In contrast, the SMPU/GNP nanocomposite, as shown in Fig. 6b, exhibits a rougher and more textured morphology, suggesting the successful incorporation of GNPs within the polymer matrix. The flake-like features indicate good dispersion and interfacial adhesion between the GNPs and SMPU, leading to enhanced surface roughness and potential improvements in mechanical properties. In contrast, the SMPU/MWCNT nanocomposite, as shown in Fig. 6c, displays a more interconnected and network-like morphology. Such morphology and strong interfacial interactions between MWCNTs and the polymer matrix enable efficient load transfer and crack bridging, thereby resulting in a marked improvement in tensile strength.

### FTIR analysis

**Fig. 7:**
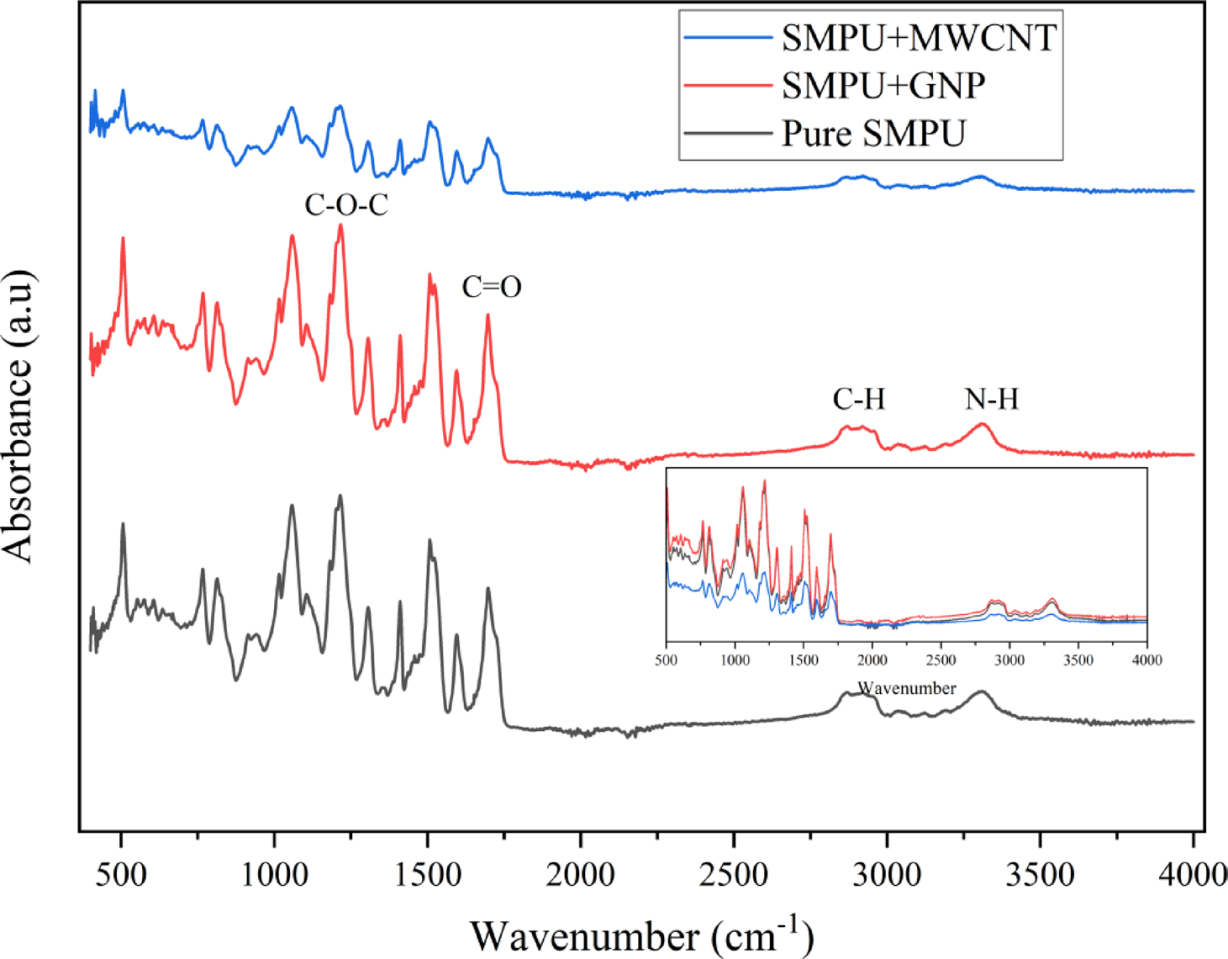
ATR-FTIR spectra analysis of SMPU and its nanocomposite films.

The chemical structures of pure SMPU and its nanocomposite films were investigated by FTIR, as illustrated in Fig. 7. A comparison of FTIR spectra of pure SMPU and GNP nanocomposite films indicates that SMPU’s characteristic peaks are still the same, which means that the addition of GNPs does not have any effect on the chemical structure of polyurethane. This also indicates that there is no chemical interaction between the functional groups of SMPU and GNP. Specifically, absorption peaks in the range between 764 and 816 cm⁻¹ are due to the out-of-plane vibrations of aromatic C-H bonds. Stretching vibrations of the C-O, C-O-C, and N-H groups are indicated at 1054, 1218, and 3306 cm⁻¹, respectively. Also, the peaks at 1509, 2864, and 2931 cm⁻¹ are attributed to N-H bending, CH₂ symmetric stretching, and CH₂ asymmetric stretching vibrations, respectively^13^.

In the carbonyl stretching area, two strong peaks are found at 1593 and 1693 cm⁻¹, which are due to hydrogen-bonded and free C = O groups, respectively^29,30^. Upon the addition of GNP, it can be concluded that GNP restricts the mobility of free carbonyl groups while enhancing the mobility of hydrogen-bonded carbonyl groups. This results in the corresponding peaks appearing at lower and higher wavenumbers, respectively, eventually causing both peaks to overlap. Furthermore, the addition of MWCNTs results in a reduction of FTIR peak intensities (particularly in N-H, C-H, and C = O regions) compared to pure SMPU and SMPU + GNP. This reduction suggests restricted mobility of SMPU functional groups, caused by strong interfacial interactions between MWCNT surfaces and urethane linkages. Diminished absorbance intensity in the hydrogen-bonding regions implies that MWCNTs hinder dipole moment variations, thereby lowering vibrational absorption signals^31,32^.

### XRD analysis

**Fig. 8:**
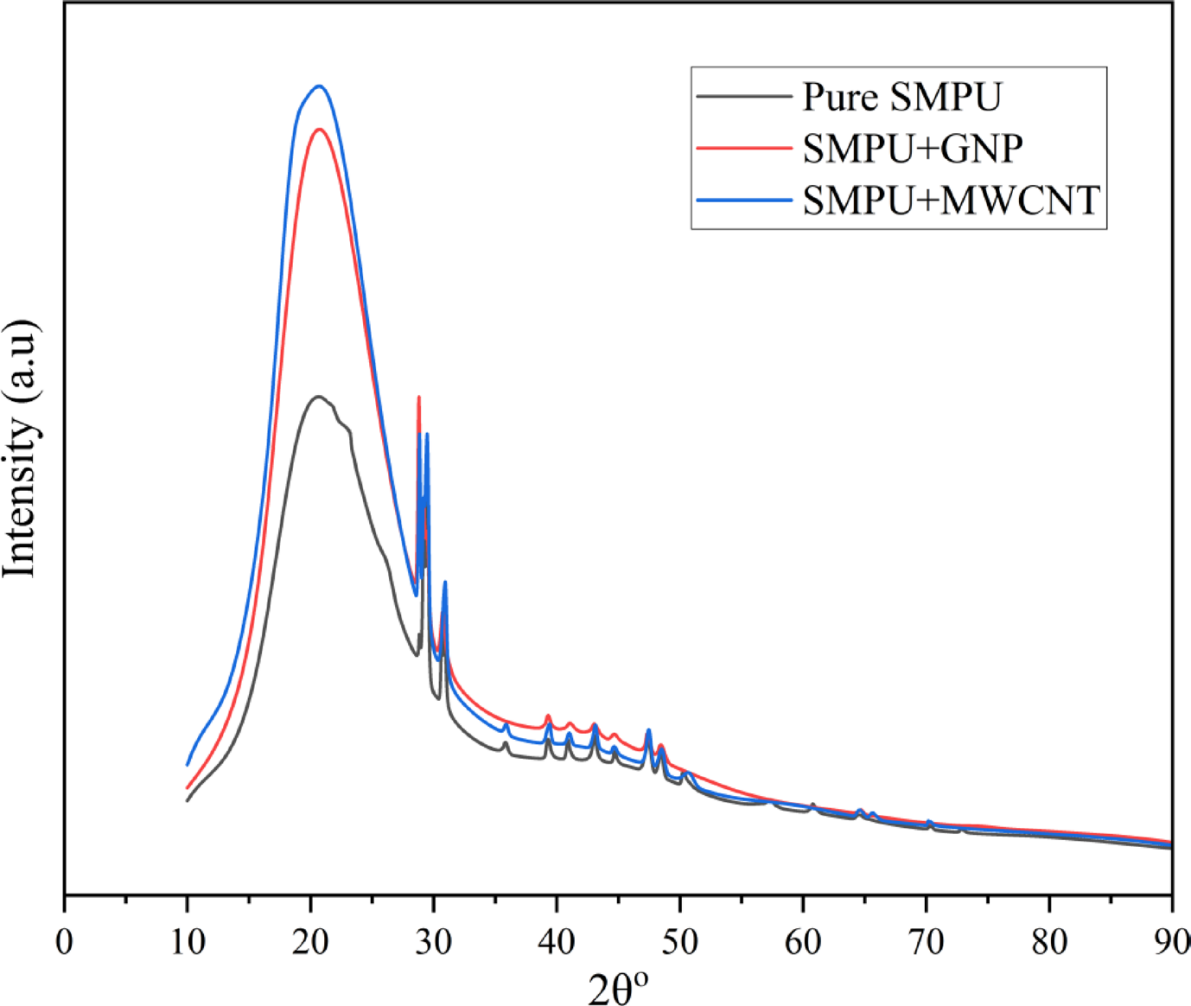
XRD Patterns of SMPU and its nanocomposite films.

Figure 8 presents the XRD patterns of pure SMPU and its nanocomposites. Pure SMPU shows a broad diffraction peak centered at 2θ = 20.66°, corresponding to the crystalline domains of the soft segments, with an overall crystallinity of 58.2%. Upon incorporation of GNPs, the relative peak areas, indicating apparent crystallinity enhanced to 64.6% due to the nucleating action of GNPs, interactions between polymer chains and GNPs, which induce better chain alignment and partial crystallinity. The SMPU/MWCNT composite exhibits an even stronger relative integrated intensity, and apparent crystallinity increased to 67.6%. This improvement is attributed to the nucleating effect of MWCNTs and their enhanced interfacial interactions with polymer chains, promoting segmental alignment and ordering^16^. The enhanced crystalline structure is expected to improve both the mechanical strength and shape-memory performance of the nanocomposites.

### Shape recovery characterization

**Fig. 9:**
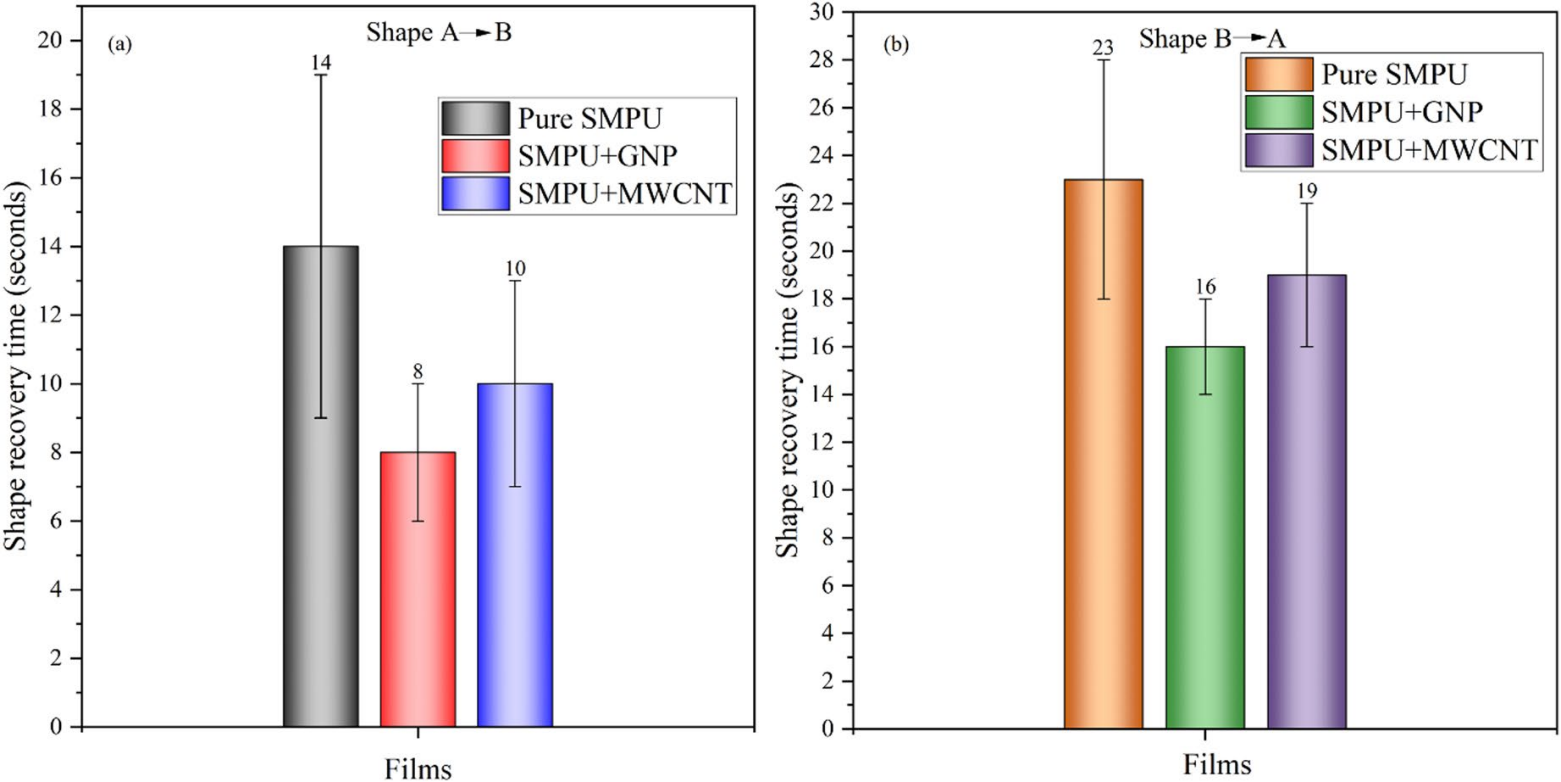
Time needed for shape recovery across all films: (**a**) A→B, (**b**) B→A.

**Fig. 10:**
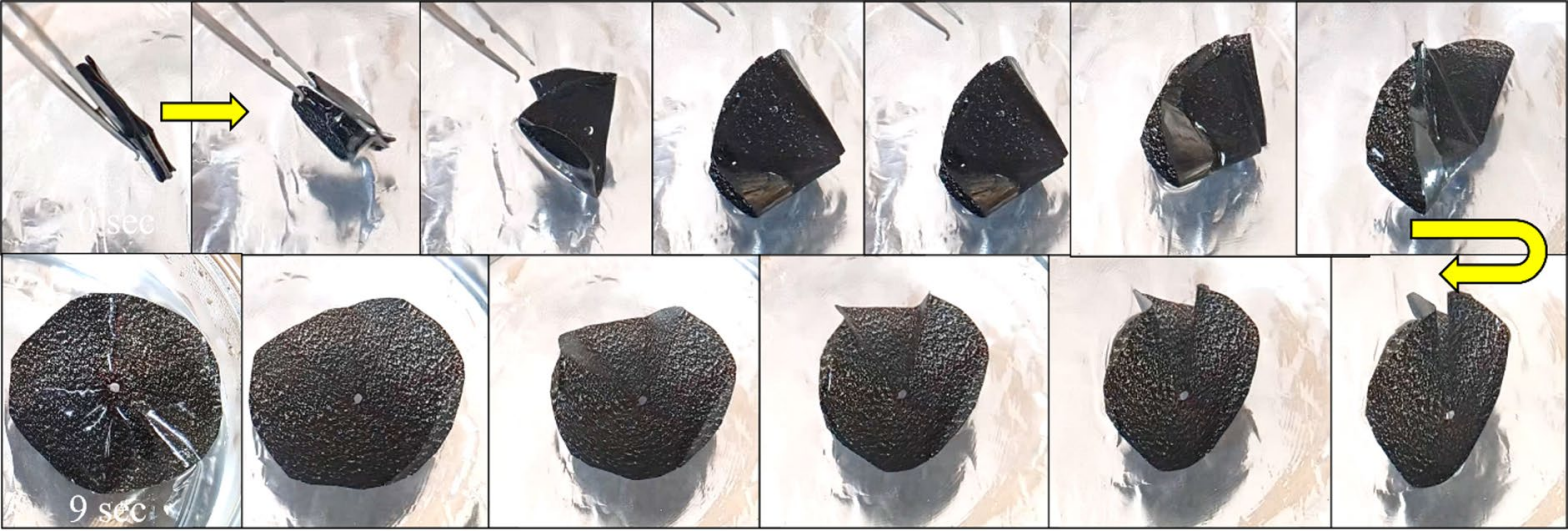
Progress of the recovery process during the transition from temporary (shape-A) to permanent (shape-B) in SMPU/MWCNT film.

Figure 9a and b illustrate the shape recovery performance of GNP and MWCNT reinforced SMPU films in A→B and B→A shape transitions. In the A→B transition, pure SMPU exhibited 100% recovery in 14 s (s), while GNP and MWCNT reinforced films recovered much faster, in 8 and 10 s, respectively. The addition of these nanofillers significantly improves the recovery behavior of SMPU films. Figure 10 shows the recovery of SMPU/MWCNT during water bath from shape A→B.

**Fig. 11:**
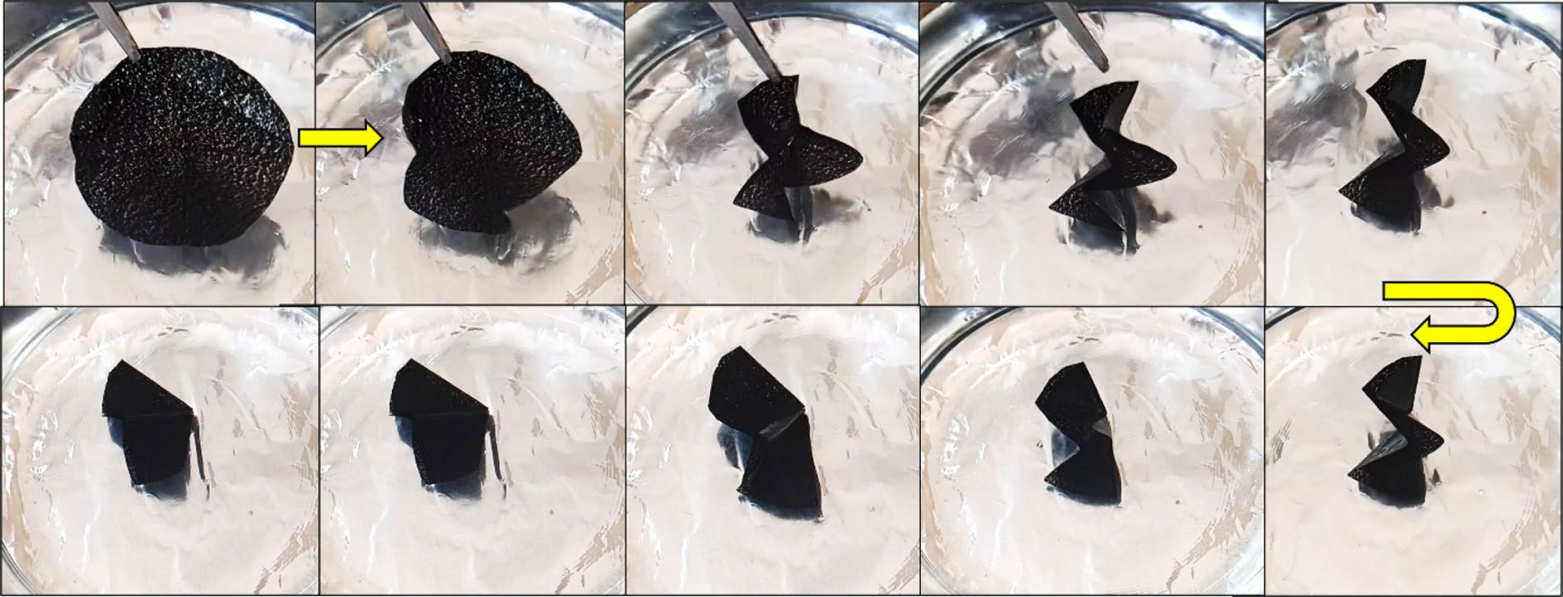
Progress of the recovery process during the transition from temporary (shape-B) to permanent (shape-A) in SMPU/MWCNT film.

This improvement can be attributed to their high thermal conductivity, which enables faster and more uniform heat transfer, allowing the films to reach the transition temperature more quickly^3,25^. Furthermore, strong interfacial interactions between the nanofillers and polymer chains facilitate stress transfer, while increased crystallinity provides stable netpoints that act as elastic anchors during recovery. Together, these mechanisms result in a sharper, faster, and more reliable recovery response in SMPU nanocomposites compared to neat SMPU. In contrast, during the B→A transition, as shown in Fig. 11, in this transition, the percentage of recovery declined to about 90% and the recovery time increased for pure SMPU 23s, SMPU/GNP 16s, and SMPU/MWCNT 19s. This slower response might be attributed to the hydrodynamic drag associated with folding in the water bath.

### Surface wettability

**Fig. 12:**
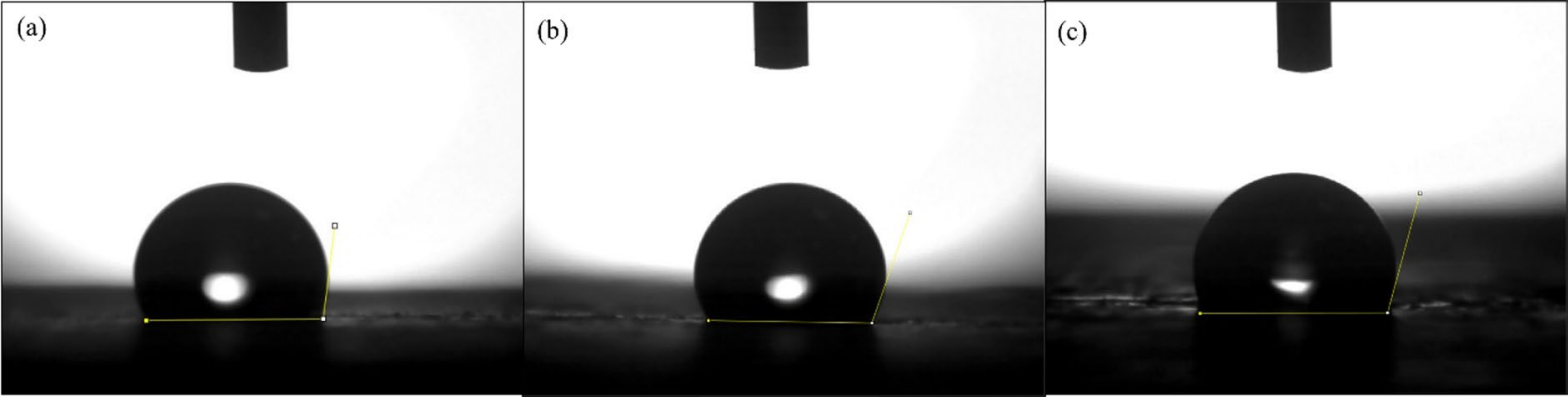
Contact angle images of (**a**) pure SMPU, (**b**) SMPU/GNPs, and (**c**) SMPU/MWCNT reinforced films.

The images of the contact angle in Fig. 12 reveal the wettability of pure SMPU and its nanocomposites. Pure SMPU, as shown in Fig. 12a, has a contact angle of 97.5°, which is moderately hydrophobic due to the balance between polar urethane groups and nonpolar soft segments. The addition of GNPs increases the contact angle to 108.3°, reflecting enhanced hydrophobicity because the graphitic surface of GNPs reduces surface energy and resists water spreading. However, the contact angle for the SMPU/MWCNT composite in Fig. 12c is 103.2°, still higher than for pure SMPU, and reflects improved hydrophobicity but slightly lower than that of the GNP composite, probably due to the presence of polar interaction sites on MWCNT surfaces.

### Effect of UV irradiation on SMPU and nanocomposite films

#### FTIR analysis after UV exposure (chemical degradation)

**Fig. 13:**
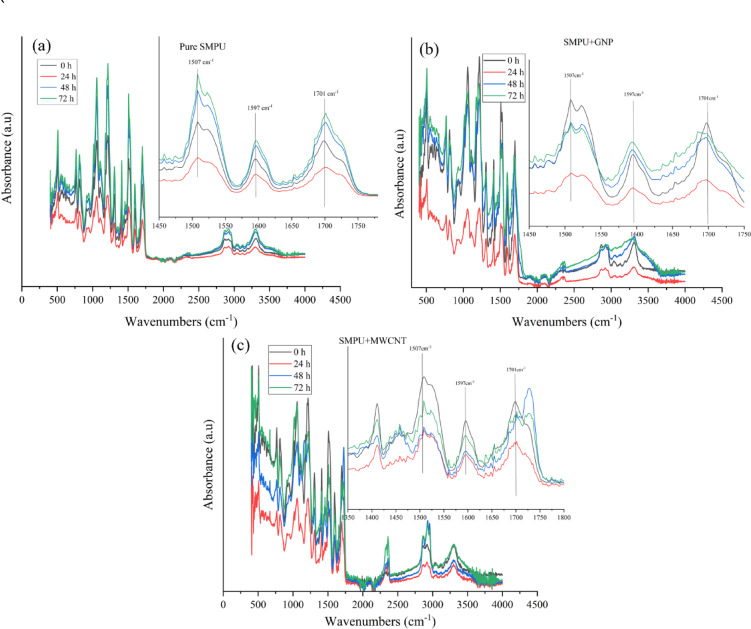
ATR-FTIR spectra analysis of SMPU and its nanocomposite films before and after 24, 48, and 72 h of UV exposure: (**a**) pure SMPU, (**b**) SMPU/GNPs, and (**c**) SMPU/ MWCNT.

The FTIR spectra in Fig. 13 show the chemical degradation behavior for three films over different irradiation time periods (0, 24, 48, 72 h): (a) Pure SMPU, (b) SMPU/GNP, and (c) SMPU/MWCNT. Considering pure SMPU (Fig. 13a) FTIR spectra reveal gradual changes over aging time across the wavenumber range (500–4500 cm⁻¹), especially in the carbonyl region (approx. 1450–1750 cm⁻¹). The area under the peaks at 1597 cm⁻¹ and 1701 cm⁻¹ decreases after 24 h of UV exposure, suggesting possible photo-crosslinking. However, it broadens and increases upon further exposure (48 h and 72 h), indicating the formation of quinone and carboxylic acid groups as a result of UV-induced photooxidation and chain scission. The carbonyl bands and aromatic C = C stretching become more pronounced with time, suggesting progressive degradation via hydrogen-bonded urethanes and photoproduct accumulation. These changes parallel photo-Fries rearrangement mechanisms as reported for aromatic polyurethane matrices^21,24^.

Considering SMPU/GNP (Fig. 13b), the area under the carbonyl and aromatic peaks decreases compared to the un-irradiated SMPU/GNP as aging progresses, while the band area at 1597 cm⁻¹, 1701 cm⁻¹, and 1730 cm⁻¹ increases over exposure time. However, the presence of GNPs appears to moderately stabilize the polymer against rapid photodegradation compared to pure SMPU. The carbonyl region’s intensity rises, but less sharply than in the pure polymer, indicating GNP may act as a UV absorber and radical scavenger, slowing overall chemical change while still allowing gradual photoproduct accumulation^21,24^.

Considering SMPU/MWCNT (Fig. 13c), the SMPU matrix containing MWCNT demonstrates similar spectral evolutions. The MWCNT nanofillers provide enhanced stabilization, reducing photoproduct formation and limiting UV-induced polymer chain cleavage. FTIR suggests the matrix with MWCNT exhibits improved resistance to chemical degradation, as evidenced by a less rapid rise in carboxylic acid and urethane photoproduct peaks over the aging period.

For quantitative analysis, the extent of peak broadening was expressed as the photooxidative index (PI), which was calculated using the following formula^24^:

2 $$\mathrm{P}\mathrm{I}={\left(\frac{A}{B}\right)}_{Exposed}-{\left(\frac{A}{B}\right)}_{Unexposed}$$

**Table 3:** Photooxidative index (PI) of SMPU and its nanocomposite films after UV exposure.

Film/specimen	UV irradiation time (h)/photooxidative index (PI)		
	24	48	72
Pure SMPU	0.200	0.286	0.341
SMPU/GNP	0.152	0.187	0.231
SMPU/MWCNT	0.124	0.146	0.196

Where A represents the area under the peak corresponding to the O-H and N-H stretching vibrations (3650 –3100 cm⁻¹), and B denotes the area under the C-H stretching peak (3000 –2700 cm⁻¹). Owing to the excellent UV absorption capability of GNPs and MWCNTs, the photooxidative index (PI) values of the nanocomposite films were lower than those of pure SMPU, though they increased gradually with prolonged UV exposure, as shown in Table 3. For pure SMPU, the PI value was 0.200, while the GNP-reinforced nanocomposites showed reduced PI values of 0.152, 0.187, and 0.231 after 24, 48, and 72 h of UV exposure, respectively. These reductions correspond to UV-resistance improvements of approximately 24, 35, and 32%, indicating the protective role of GNPs. Similarly, the MWCNT-based films exhibited PI values of 0.124, 0.146, and 0.196 at the same exposure durations, indicating UV-resistance enhancements of 38, 49, and 42%. Moreover, the gradual decrease in PI values with increasing exposure time suggests that the nanofillers suppress excessive photo-oxidation, although prolonged irradiation may still trigger higher levels of oxidation.

Furthermore, the progressive increase in PI (pure SMPU) with irradiation time correlates directly with the visual yellowing observed in Fig. 3. In aromatic polyurethanes, UV exposure induces photochemical rearrangement and formation of conjugated carbonyl and quinone-type chromophores, which absorb in the visible region and produce yellow discoloration. The absorption band at 1507 cm⁻¹ corresponds to the N-H bending vibration of the amide bond. In pure SMPU, an increase in the intensity of this peak as compared to unirradiated was associated with the progression of photooxidation. In contrast, the lower PI values observed for SMPU/GNP and SMPU/MWCNT films indicate reduced chromophore formation, confirming enhanced UV resistance imparted by the graphitic nanofillers^33,34^. Moreover, the nanocomposite films exhibited minimal change in this peak intensity, confirming their enhanced UV resistance and reduced photooxidative degradation compared to pure SMPU. These nanotubes absorb UV light, stabilize radicals through π-electron delocalization, and hinder oxygen diffusion within the polymer matrix, thereby suppressing photooxidative degradation^35,36^.

Overall, across all films, FTIR spectra show that polymer matrices undergo photooxidation, photoproduct formation, and cross-linking during UV exposure. Nanofillers (GNP or MWCNT) systematically reduce the rate and extent of these chemical changes, supporting their role in improving polymer durability.

#### Thermal behavior (DSC after UV)

**Fig. 14:**
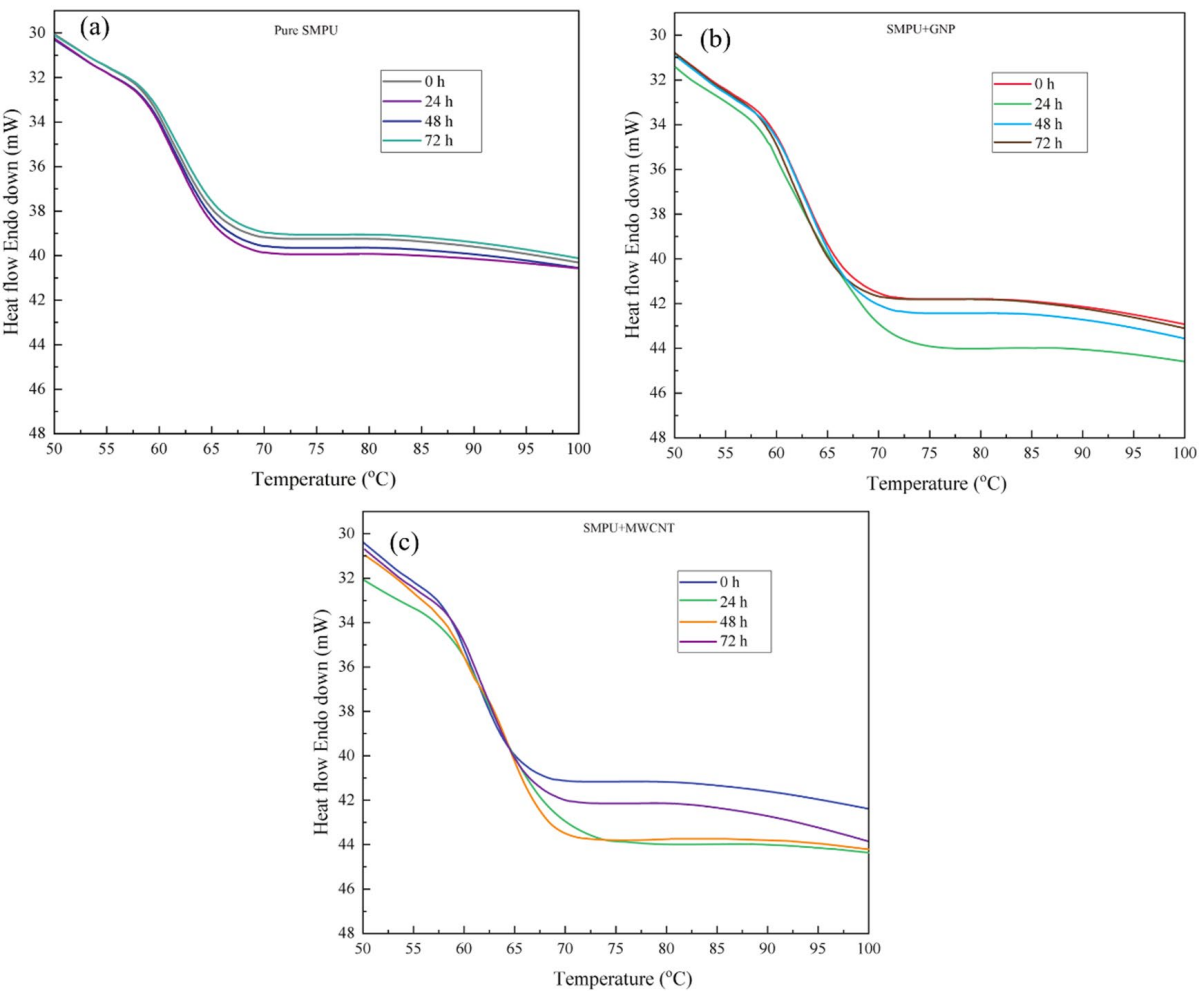
DSC thermograms of SMPU and its nanocomposite films before and after 24, 48, and 72 h of UV exposure: (**a**) pure SMPU, (**b**) SMPU/GNPs, and (**c**) SMPU/ MWCNT.

The DSC thermograms of pure SMPU, SMPU/GNP, and SMPU/MWCNT nanocomposite films before and after UV exposure are presented in Fig. 14a–c. For pure SMPU (Fig. 14a), a noticeable shift of the T_g_ toward higher values was observed after initial UV exposure. The T_g_ increased from 63.5 to 67.2 °C after 24 h, which can be attributed to photo-crosslinking reactions and secondary ordering within the hard segment domains. These processes restrict the mobility of the soft segments and enhance the overall rigidity of the polymer network^23^.

However, with prolonged irradiation (48 h and 72 h), the T_g_ declined to 63.4 °C and 62.5 °C, respectively. This decrease indicates that chain scission reactions begin to dominate over crosslinking beyond a critical irradiation time^23^. Ultraviolet photons possess sufficient energy to cleave urethane linkages and C-O/C-N bonds, resulting in molecular degradation. The fragmentation of polymer chains increases free volume and enhances segmental mobility, thereby lowering the T_g_. In the SMPU/GNP nanocomposite (Fig. 14b), the T_g_ remained consistently higher than that of pure SMPU throughout the entire exposure period, confirming the reinforcing and stabilizing effect of GNPs. With increasing UV exposure, the T_g_ followed the trend 66.4 °C → 68.7 °C → 65.5 °C → 64.6 °C (0–72 h). This behavior can be attributed to strong filler-polymer interfacial interactions that hinder chain relaxation and delay soft-segment motion. Moreover, the decrease in T_g_ after 48 h of UV exposure is minimal and appears to stabilize, likely because the GNPs act as UV barriers, reducing photodegradation and promoting limited crosslinking rather than chain scission^37^.

Similarly, the SMPU/MWCNT nanocomposite, Fig. 14c, on the other hand, presented T_g_ values of 65.6 °C → 67.3 °C → 66.0 °C → 65.9 °C with increasing exposure time. These gradual shifts of T_g_ to a slightly higher temperature are indicative of increased thermal stability and restricted chain mobility. Strong π–π interactions between MWCNTs and the urethane backbone ensure proper dissipation of UV energy and therefore prevent extensive degradation. Furthermore, MWCNTs enhance localized heat distribution and physical crosslinking, which also stabilizes the polymer matrix.

The overall DSC results demonstrate that the improved thermal stability of the nanocomposite films under UV exposure is primarily attributed to the UV shielding effect of graphitic nanofillers. Due to their conjugated π-electron structures, GNPs and MWCNTs effectively absorb UV radiation at 340 nm, thereby reducing photon penetration into the polymer matrix and limiting photoinitiated bond cleavage. While radical stabilization through π-electron delocalization and oxygen diffusion barrier effects may contribute, UV shielding is considered the dominant mechanism at the present filler loading (1 wt%). This reduced photoinitiation explains the smaller Tg fluctuations observed in nanocomposite films compared to pure SMPU.

#### Mechanical behavior after UV exposure

**Fig. 15:**
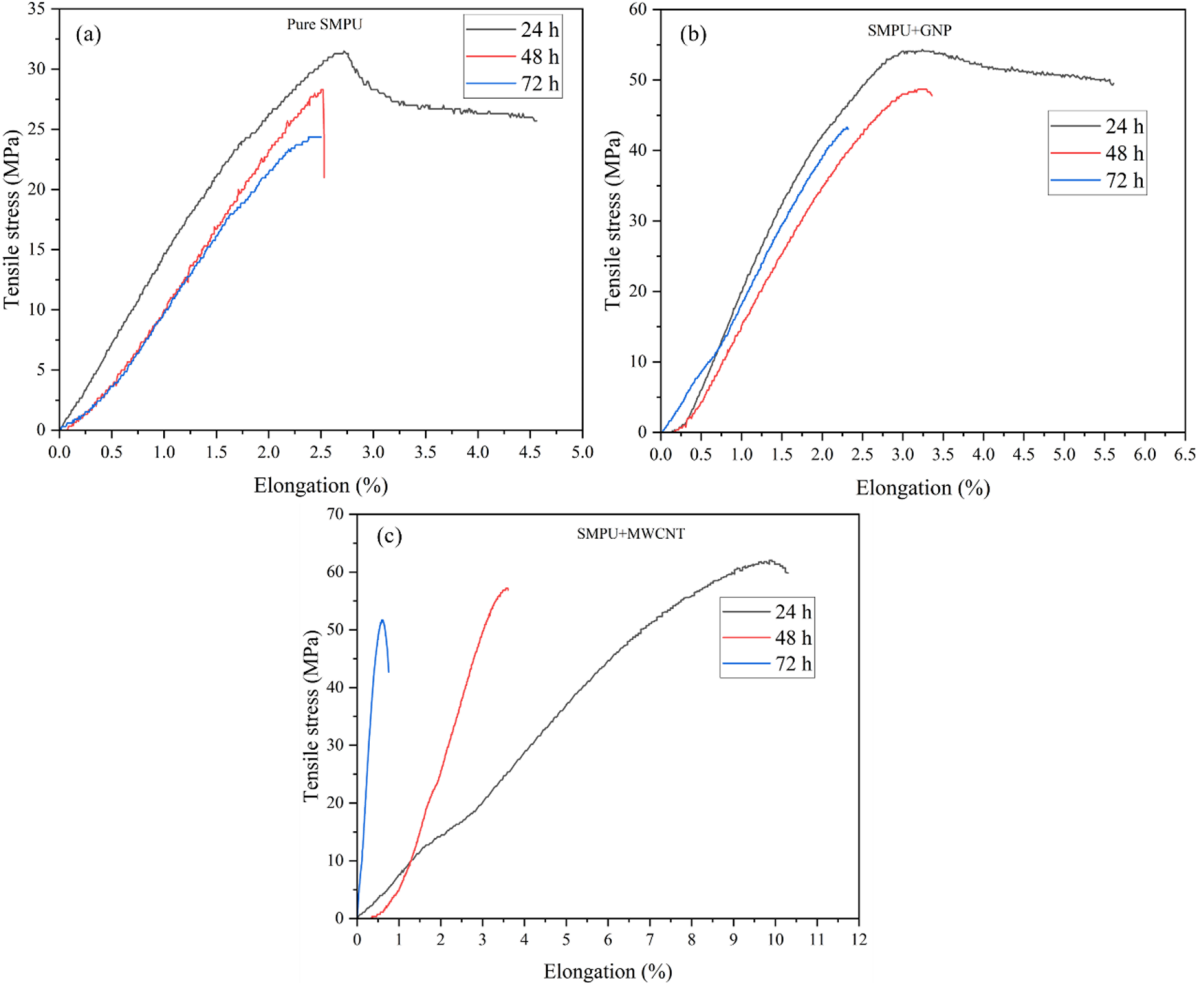
Tensile stress/elongation of SMPU and its nanocomposite films after 24, 48, and 72 h of UV exposure: (**a**) pure SMPU, (**b**) SMPU/GNPs, and (**c**) SMPU/MWCNT.

The tensile stress-elongation curves for pure SMPU, SMPU/GNP, and SMPU/MWCNT nanocomposites after UV exposure are presented in Fig. 15. Before UV irradiation, all films exhibited good ductility and moderate tensile strength. However, exposure to UV light induced competing effects of photo-crosslinking, chain scission, and photo-oxidation, which collectively governed the mechanical response over time^23^. For pure SMPU (Fig. 15a), the tensile strength initially increased from 17 to 32 MPa after 24 h of exposure, attributed to photo-crosslinking within the polymer matrix. This temporary improvement was followed by a sharp decline to 22 MPa at 48 h and 14 MPa at 72 h, as chain scission and oxidative degradation became dominant. Prolonged irradiation also caused visible discoloration (yellowing) as shown in Fig. 3, a pronounced reduction in elongation, and overall embrittlement of the material^21,23^.

**Fig. 16:**
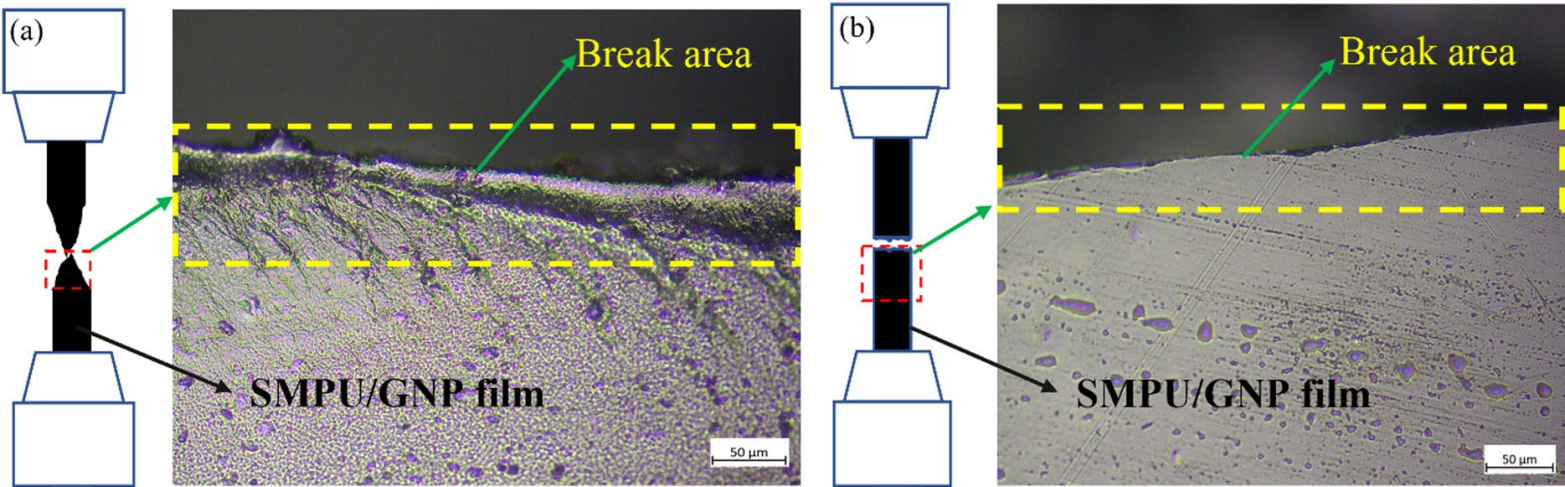
Optical micrographs of the SMPU/GNP film after tensile testing: (**a**) before UV exposure and (**b**) after UV exposure.

The SMPU/GNP (Fig. 15b), nanocomposite initially exhibited a high tensile strength of 55.3 MPa at 24 h, followed by gradual reductions to 48.7 MPa (48 h) and 43.2 MPa (72 h). Although the GNP-reinforced film retained higher strength than pure SMPU at all times, it also experienced a noticeable loss of ductility with extended exposure. After 24 h, the films became increasingly brittle and fractured sharply during tensile testing, as shown in Fig. 16. A comparison of fracture surfaces (Fig. 16a & b) reveals a clear change in the failure mode: before UV exposure, the film displayed a rough surface and drawn-out fracture characteristic of ductile behavior, whereas after UV exposure (72 h), the surface became smoother, indicative of brittle failure induced by UV degradation.

Similarly, the SMPU/MWCNT (Fig. 15c), film exhibited superior stability, maintaining 62 MPa (24 h), 56.1 MPa (48 h), and 51.7 MPa (72 h). This stability is attributed to the unique network structure and mechanical flexibility of MWCNTs, which not only absorb UV light and dissipate mechanical loads but also crack bridging and defects generated during irradiation.

Overall, the incorporation of nanofillers acted as both physical barriers to UV penetration and chemical stabilizers against radical-induced reactions, thereby reducing the rate of photo-oxidative damage and preserving the mechanical integrity of the SMPU matrix over extended UV exposure, although progressive degradation remained unavoidable^33,34^.

#### XRD analysis after UV exposure (structural)

**Fig. 17:**
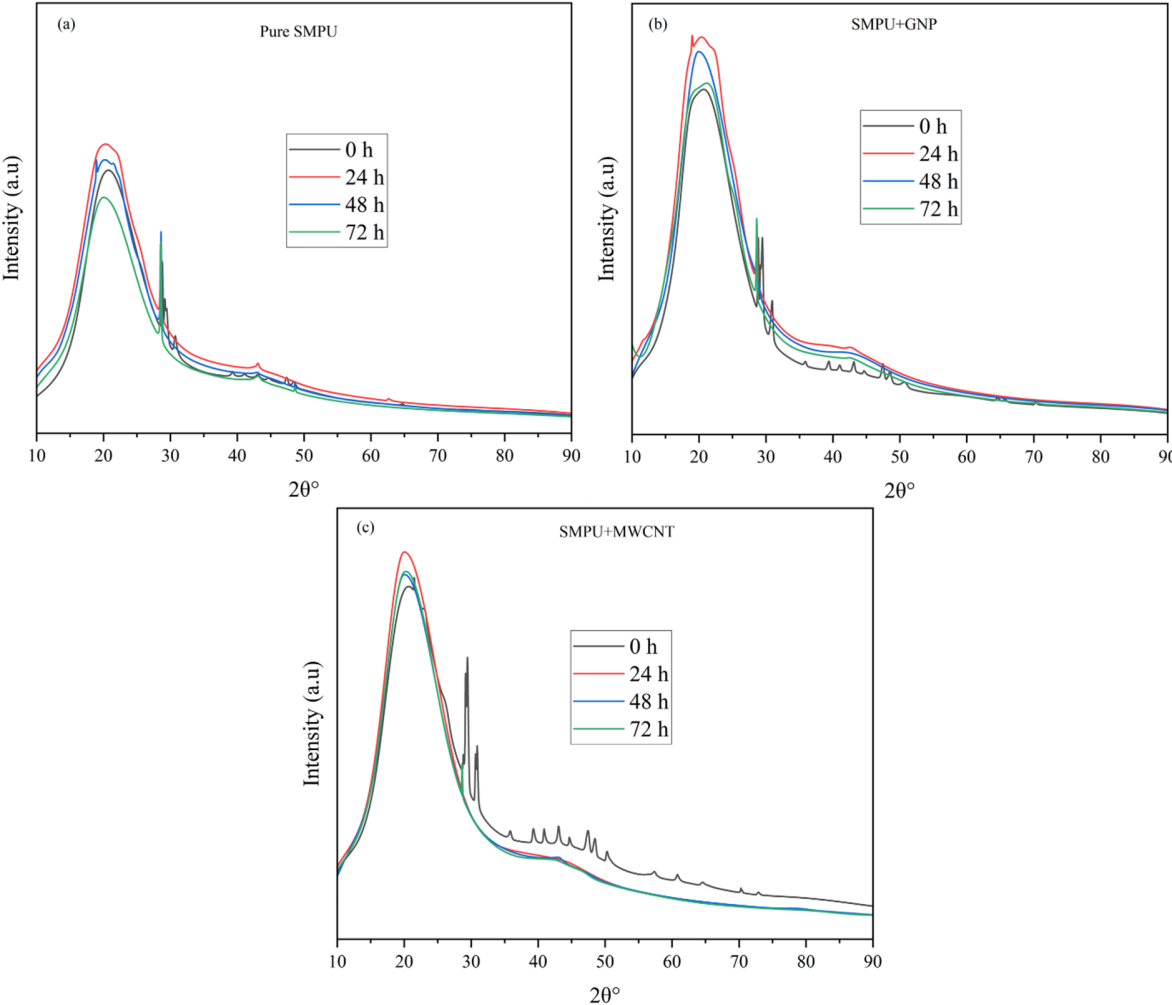
XRD Patterns of SMPU and its nanocomposite films before and after 24, 48, and 72 h of UV exposure: (**a**) pure SMPU, (**b**) SMPU/GNPs, and (**c**) SMPU/ MWCNT.

The XRD patterns of pure SMPU, SMPU/GNP, and SMPU/MWCNT nanocomposite films before and after UV irradiation are shown in Fig. 17. Upon UV irradiation, the diffraction peaks of all films showed slight intensity changes and peak broadening, reflecting alterations in crystalline order due to photochemical reactions. The percentage of crystallinity index is displayed in Table 4.

**Table 4:** Crystallinity index (CI) (%) of SMPU and its nanocomposite films before and after UV exposure.

Film/specimen	UV irradiation time (h)/crystallinity index (CI) (%)			
	0	24	48	72
Pure SMPU	58.2	62.8	59.4	57.8
SMPU/GNP	64.6	67.4	66.1	65.3
SMPU/MWCNT	67.6	71.6	69.4	68.3

For pure SMPU (Fig. 17a), CI initially increased to 62.8% after 24 h, attributed to photo-crosslinking and secondary ordering in the hard segment domains. However, prolonged exposure (48 h and 72 h) reduced CI to 59.4 and 57.8%, respectively, as chain scission and oxidation disrupted crystalline packing and destroyed ordered domains. In contrast, SMPU/GNP (Fig. 17b) retained higher crystallinity throughout exposure, with CI values of 67.4% (24 h), 66.1% (48 h), and 65.3% (72 h). The GNPs act as physical barriers to UV penetration, minimizing polymer chain fragmentation and maintaining structural order. Similarly, SMPU/MWCNT (Fig. 17c) showed exceptional stability with CI values of 71.6% (24 h), 69.4% (48 h), and 68.3% (72 h), indicating that the nanotube network effectively restricts molecular motion and resists photodegradation. The minimal decline in crystallinity for the nanocomposites compared to the sharp reduction in pure SMPU confirms that both GNPs and MWCNTs significantly suppress UV-induced disruption of the crystalline domains.

#### Surface wettability after UV exposure (surface changes)

**Fig. 18:**
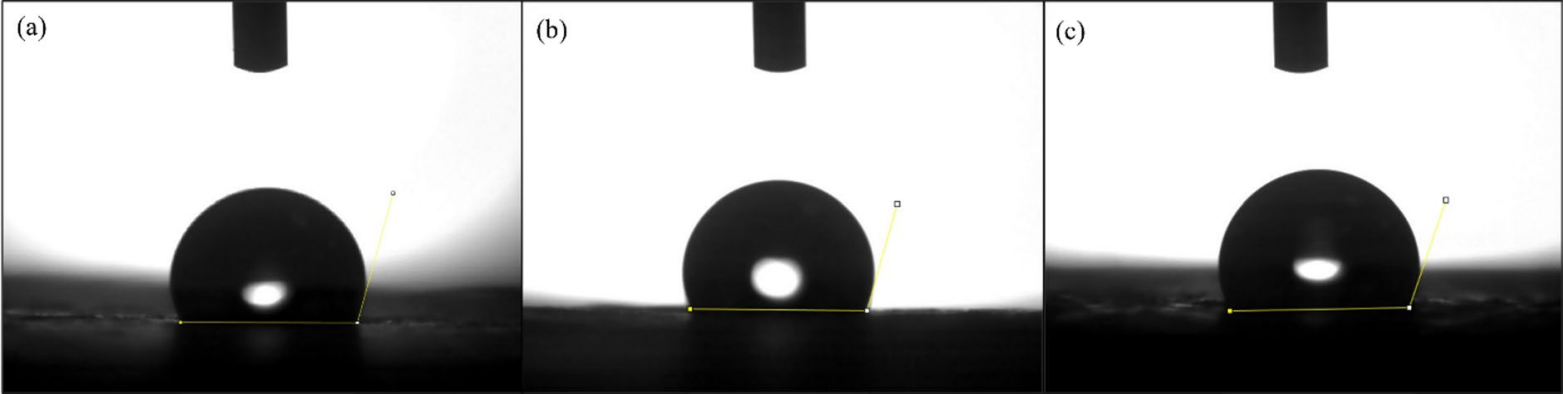
Contact angle analysis of SMPU and its nanocomposite films after 24 h of UV exposure: (**a**) pure SMPU, (**b**) SMPU/GNP, and (**c**) SMPU/ MWCNT.

The contact angle measurements of pure SMPU, SMPU/GNP, and SMPU/MWCNT films after UV irradiation for 24 h are shown in Fig. 18, with the corresponding numerical values before and after UV summarized in Table 5. All films exhibited an initial increase in contact angle after 24 h of UV exposure; however, with prolonged irradiation for 48 and 72 h, a gradual decrease in contact angle occurred. Considering the pure SMPU film, the contact angle increased from 97° (un-irradiated) to 105.3° after 24 h, due to promoting surface rearrangement and migration of low-energy soft-segment chains (such as aliphatic urethane or ether groups) toward the air interface. This reorganization reduces surface energy, leading to an increase in contact angle and enhanced hydrophobicity. However, further irradiation for 48 h and 72 h reduced the contact angle to 103.4° and 99.7°, respectively, resulting in the formation of polar oxygen-containing functional groups (-OH, -C = O, -COOH) on the surface. These newly formed hydrophilic moieties increase surface polarity and roughness, thereby lowering the contact angle and enhancing water wettability.

**Table 5:** Values of the contact angle of SMPU and its nanocomposite films before and after UV exposure.

Film/specimen	UV irradiation time (h)/ contact angle (°)			
	0	24	48	72
Pure SMPU	97.5	105.3	114.5	110.1
SMPU/GNP	108.3	103.4	111.6	108.3
SMPU/MWCNT	103.2	99.7	108.2	105.2

A similar trend was observed for the nanocomposite films. The SMPU/GNP sample showed the highest hydrophobicity, with the contact angle increasing to 114.5° at 24 h and then decreasing to 111.6° and 108.2° at 48 h and 72 h, respectively. The SMPU/MWCNT film exhibited contact angles of 110.1°, 108.3°, and 105.2° at corresponding irradiation times. The higher contact angles in nanocomposites compared to pure SMPU can be attributed to the presence of graphitic nanofillers that contribute to lower surface energy. The incorporation of graphitic nanofillers (GNPs and MWCNTs) mitigates these degradation effects by absorbing and dissipating UV energy, thus maintaining higher surface hydrophobicity and structural stability compared to the pure SMPU film.

#### Shape recovery behavior after UV exposure (functional performance)

The thermally induced shape recovery performance of pure SMPU and its nanocomposite films was examined in both Shape A→B (folded-to-unfolded) and Shape B→A (unfolded-to-folded) transitions after UV irradiation for 24, 48, and 72 h. After UV exposure, the A→B recovery time of pure SMPU is quickly recovered within 11 s at 24 h, 13 s (48 h), and 18 s (72 h), with a recovery ratio drop at 72 h from 100% to approximately 95%. In the B→A transition, the deterioration was more pronounced; recovery times extended to 19 s, 31 s, and 36 s over the same exposure durations, and recovery ratios decreased to 90%. The decline results from UV-induced chain scission and oxidation of the soft segments, which weaken elastic recovery and hinder segmental rearrangement during refolding.

In contrast, both nanocomposite films exhibited better retention of recovery efficiency. After 72 h of UV irradiation, SMPU/GNP and SMPU/MWCNT maintained A→B recovery ratios of 100% and B→A ratios around 90%, with only moderate increases in recovery time. This stability is attributed to the nanofillers role in dissipating UV energy, restricting segmental damage, and preserving reversible phase domains responsible for shape-memory behavior.

Overall, the results confirm that the incorporation of GNPs and MWCNTs not only accelerates shape recovery but also preserves recovery efficiency under prolonged UV irradiation, highlighting their stabilizing role in maintaining the functional integrity of SMPU-based smart materials.

## Conclusions

Thin-films of pure SMPU and its nanocomposites reinforced with 1wt% GNPs and MWCNTs were successfully fabricated using the solvent casting method and comprehensively characterized for their thermal, mechanical, and shape recovery and UV-resistant properties with and without UV irradiation. While enhanced UV resistance of graphitic nanofillers is consistent with prior literature, the present study demonstrates that such stabilization directly contributes to the preservation of shape memory performance under prolonged UV exposure. The nanocomposite films retained higher recovery ratios and faster actuation compared to neat SMPU, highlighting the importance of UV stabilization in maintaining functional reliability of SMP-based thin-film devices. The following conclusions can be drawn:

- T_g_ of SMPU nanocomposite films increased as a result of restricted chain mobility induced by the nanofillers.
- The tensile properties were significantly enhanced due to effective filler–matrix interactions and efficient load transfer.
- The reinforced films exhibited faster shape recovery compared to pure SMPU, which can be attributed to the enhanced thermal conductivity imparted by the incorporated GNPs and MWCNTs.
- After 24 h of UV irradiation, the mechanical, thermal, and shape recovery properties exhibited improvement, attributed to the photo-crosslinking mechanism.
- Prolonged UV irradiation (up to 72 h), pure SMPU exhibited chain scission, discoloration, and embrittlement due to photo-degradation.
- FTIR confirmed reduced formation of oxidative photoproducts in nanocomposites after UV exposure due to the nanofillers acted as UV shields and radical scavengers.
- Overall, the incorporation of GNPs and MWCNTs enhanced the multifunctional behavior of SMPU thin-films, offering improved durability, flexibility, and UV resistance.

## Data Availability

Data is provided within the manuscript.
